# Evaluation of an Intervention to Promote Self-Management Regarding Cardiovascular Disease: The Social Engagement Framework for Addressing the Chronic-Disease-Challenge (SEFAC)

**DOI:** 10.3390/ijerph192013145

**Published:** 2022-10-12

**Authors:** Sophie A. Korenhof, Ellen V. Rouwet, Liset E. M. Elstgeest, Siok Swan Tan, Stefania Macchione, Vanja Vasiljev, Tomislav Rukavina, Tamara Alhambra-Borrás, Irene N. Fierloos, Hein Raat

**Affiliations:** 1 Department of Public Health, Erasmus University Medical Center, 3015 GD Rotterdam, The Netherlands; 2 Reinier Academy, Reinier de Graaf Hospital, 2625 AD Delft, The Netherlands; 3 Research Group City Dynamics, InHolland University of Applied Sciences, 3072 AG Rotterdam, The Netherlands; 4 European Project Office Department, Istituto per Servizi di Ricovero e Assistenza agli Anziani (Institute for Hospitalization and Care for the Elderly), 31100 Treviso, Italy; 5 Department of Social Medicine and Epidemiology, Faculty of Medicine, University of Rijeka, 51000 Rijeka, Croatia; 6 Polibienestar Research Institute, University of Valencia, 46022 Valencia, Spain

**Keywords:** mindfulness, cardiovascular disease, risk factors, self management, chronic disease management

## Abstract

Background: Cardiovascular diseases (CVD) are predominantly lifestyle related. Mental health issues also influence CVD progression and quality of life. Self-management of lifestyle behaviors and mental well-being may play a significant role in reducing the CVD burden. Previous studies have shown that mindfulness practices are associated with psychological well-being, but their effects on CVD self-management are mainly unknown. Methods: The study had a before–after design and included adults over 50 years with CVD and/or one or more risk factors from three European countries. Follow-up was six months. The intervention was a 7-week mindfulness-based intervention (MBI) in a group setting focusing on chronic disease self-management. Outcomes were measured with validated self-report questionnaires at baseline and follow-up: self-efficacy, physical activity, nutrition, smoking, alcohol use, sleep and fatigue, social support, stress, depression, medication adherence, and self-rated health. Results: Among 352 participants, 324 (92%) attended ≥4 of the 7 group sessions and completed follow-up. During follow-up, self-efficacy, stress, social support, depressive symptoms, and self-rated health significantly improved. No significant changes were detected for other outcomes. Conclusions: A 7-week MBI focusing on chronic disease self-management was conducive to improved self-efficacy, emotional well-being, social support, and self-rated overall health during six months. These findings support the use of MBIs for improving self-management in cardiovascular care. ISRCTN registry-number ISRCTN11248135.

## 1. Introduction

Despite substantial improvements in outcomes in recent decades, cardiovascular diseases (CVD) are still the leading causes of morbidity and mortality globally, with an estimated 17 million deaths each year [1]. 

Much of the global burden of CVD is attributable to uncontrolled behavioral risk factors, including poor diet, physical inactivity, and smoking [2]. In addition, psychological distress, such as chronic stress, depression, anxiety, and post-traumatic stress disorder, contributes to poorer quality of life and increases the risk of cardiovascular events and all-cause mortality [3,4]. Conversely, favorable lifestyle behaviors and psychological well-being promote cardiovascular health [5]. For the primary and secondary prevention of atherosclerotic vascular disease, heart failure, atrial fibrillation, and other cardiovascular conditions, lifestyle behaviors and mental health are of great importance [6]. Since patients spend very little time with healthcare providers, individuals largely depend on self-care to promote physical and emotional health and manage chronic illness. Despite the importance and effectiveness of self-care in preventing and managing CVD, many individuals find it challenging to make enduring modifications in lifestyle behaviors, take care of their mental health, and deal with chronic conditions [7,8]. Programs to enhance self-management skills based on behavioral interventions are only modestly effective, and novel intervention targets are needed to improve their impact [9]. 

One such intervention target is mindfulness, commonly defined as moment-to-moment awareness of one’s experience without judgement [10]. Mindfulness is considered a metacognitive process that enhances the capacity for self-regulation, i.e., adaptively regulating one’s attention, emotions, cognition, and behavior to respond effectively to internal and external demands. Mindfulness is deeply rooted in ancient Eastern philosophies and has received considerable public interest in recent decades. This universal human capacity can be strengthened through meditation, mind–body practices, such as yoga, and the application of mindful attention in daily life. A growing body of evidence indicates that mindfulness-based interventions (MBIs) can help people cope across a broad range of medical and psychological conditions, including depression, stress, anxiety, and chronic pain [11,12]. MBIs are commonly offered as secular, manualized, group-based interventions, the most popular of which are mindfulness-based stress reduction (MBSR) and mindfulness-based cognitive therapy. Typically, a package of mindfulness practices is provided for eight weeks, including body scan, sitting meditation, walking meditation, and gentle yoga exercises. Although mindfulness practices also produce relaxation in the body, relaxation is not the primary objective. Instead, these practices teach participants to focus on present-moment experiences with an orientation of openness, kindness, curiosity, and acceptance instead of ruminating about the past or worrying about the future. By cultivating an even-minded, witnessing relationship with (distressing) physical sensations, emotions, and thoughts as passing events arising in the body-mind system, practitioners learn to undo automatic habitual responses, counter negative thought patterns, and increase cognitive flexibility. Through modulation of attention control, emotion regulation, self-awareness, motivation and learning, mindfulness is postulated to influence self-regulation and behaviors that affect physical or mental health and quality of life [13].

Although preliminary research suggests that mindfulness may influence blood pressure and glycemic control, the potential of mindfulness training in facilitating self-management of CVD has received limited attention [14]. Therefore, the Social Engagement Framework for Addressing the Chronic-disease-challenge (SEFAC) study aimed to examine the effects of the 7-week SEFAC intervention targeting health behaviors and psychosocial factors for improving self-management in adults over 50 with or at increased risk of CVD.

## 2. Methods

### 2.1. Study Design

The SEFAC study had a before–after single-group design and was conducted between 28 November 2018 and 26 October 2020 in 4 European countries (Croatia, Italy, United Kingdom (UK), and the Netherlands). For full details of study design and protocol, see Zhang et al. [15] ISRCTN registry number is ISRCTN11248135, the date of registration is 30 August 2018 (retrospectively registered). Ethics approval was provided by the human research ethics boards of the study sites [15]. All participants provided written informed consent. This article reports on health outcomes as assessed at six months follow-up. We used the SPIRIT reporting guidelines [16].

### 2.2. Recruitment and Eligibility Criteria

Recruitment for participation in a study investigating a new intervention to improve the self-management of chronic cardiovascular diseases through mindfulness training, social engagement, and e-health support was conducted in the study sites (located in Rijeka, Croatia; Treviso, Italy; Camborne, United Kingdom; and Rotterdam, the Netherlands). Community-dwelling citizens were recruited using public events and announcements, patient and volunteer organizations, and social media. Main eligibility criteria were age over 50 years and established CVD (i.e., ischemic heart disease, cerebrovascular disease, peripheral artery disease, heart failure, or other cardiovascular conditions) and/or one or more risk factors for the development of CVD (including diabetes, smoking, hypertension, hypercholesterolemia, obesity, positive family history). In addition, citizens were not eligible to participate in the study when they were diagnosed with mild or severe cognitive impairment, were terminally ill or scheduled to enter secondary or tertiary care settings for a long period, not able to comprehend the information provided in the local language, or not able to make an informed decision regarding participation in the study. 

### 2.3. Intervention

The SEFAC intervention to promote health behaviors and psychological well-being had a duration of 7 weeks, and consisted of three elements, (i) weekly mindfulness-based sessions for seven weeks, (ii) support by social engagement, and (iii) e-health support by the SEFAC app [15]. 

Ad (i): The mindfulness-based sessions were offered in a series of seven weekly in-person group sessions (11–12 participants), lasting 2–2.5 h each for which a workbook was available. The sessions focused on mindset, habit change, relationships, and living with chronic conditions and included elements of positive psychology and health coaching in addition to mindfulness training. They were conducted by certified mindfulness professionals and/or health care professionals with training in mindfulness. Furthermore, local volunteers facilitated the intervention during and between the sessions in terms of logistics, raising awareness for self-management, motivating participants to commit to the intervention, and assisting them with e-health support. The intervention incorporated the core elements of the standard MBSR protocol: (a) mental and physical mindfulness exercises (body scan, sitting meditation, walking meditation, gentle yoga exercises; Supplement Table S1; (b) how to apply mindfulness to everyday situations and stress management; (c) sharing of experiences of mindfulness and insights into automatic patterns and habits among participants. During the sessions, participants were trained to foster greater awareness of present moment experience to promote mental well-being, build self-efficacy, enhance quality of life, adopt a healthy lifestyle, and connect to their community. 

Ad (ii): Social engagement was used to support the mindfulness-based sessions [17]. Volunteers facilitated the organization and the sessions (see above). The group sessions provided an environment for peer-to-peer support allowing participants to share their experiences, offer encouragement, and share advice. Group factors such as normalizing one’s experience, a sense of shared group identity, and the empathic and anxiety-relieving nature of the group setting are considered helpful [18]. 

Ad (iii): In addition to the weekly sessions, participants were encouraged to engage in the practices (described in Supplement Table S1) and reflections in between the sessions using e-health support, consisting of the free SEFAC app developed for the Android operating system. The app included audio recordings of the mindfulness practices, tips and reflections on nutrition, stress reduction, emotional health, and physical activity. In addition, it allowed the user to set personal goals to support habit change. Participants were encouraged to download the app on their mobile phone or tablet at the start of the SEFAC intervention and use it as digital support during the seven weeks of mindfulness-based sessions; it should be noted that the app stayed available to be used without support and on a voluntary basis until six months after the start of the intervention. 

### 2.4. Outcome Measures

Outcomes were obtained through self-report questionnaires at the start of the first session (baseline) and at 6-month follow-up to assess the ‘mid-term’ effects (i.e., the sustainability of the effects four months after finishing the intervention), as suggested by Ory et al. and Barkan et al. [19,20]. The internal consistency was assessed for each multi-item scale by computing Cronbach’s alphas, which was considered sufficient when over 0.70, and preferably under 0.95 [21]. The degree of self-efficacy was determined with four instruments: Self-Efficacy for Managing Chronic Diseases 6-item scale (SEMCD-6), Cronbach’s alpha 0.88 General Self-efficacy Scale (GSES), Cronbach’s alpha 0.91; Physical Exercise Self-Efficacy Scale (PESES), Cronbach’s alpha 0.94; Nutrition Self-Efficacy Scale (NSES), Cronbach’s alpha 0.95, with higher values indicating more self-efficacy [22,23,24]. Health behaviors were assessed in six domains, in line with the American College of Lifestyle Medicine [25]: (1) Healthy eating: three items on the intake of fruit and vegetables as well as having breakfast; (2) Physical activity: six items on physical exercise [26]; one item on sedentary behavior: International Physical Activity Questionnaire (IPAQ) [27]; (3) Substance use: current smoking, yes/no; frequency of alcohol use, one item from the AUDIT-C [28]; (4) Stress management: Perceived Stress 10-item Scale (PSS-10), range 0–40, Cronbach’s alpha 0.86, with higher values indicating more stress, score <14 corresponds to low stress and score ≥14 to moderate or high perceived stress [29]; (5) Sleep and fatigue: visual analogue scales (VAS), range 0–10, with higher values indicating worse sleep/more fatigue); (6) Relationships: Oslo Social Support 3-item scale (OSSS-3), Cronbach’s alpha 0.54 [30]. Medication adherence was assessed with the Simplified Medication Adherence Questionnaire (SMAQ), Cronbach’s alpha 0.57 [31]. Depression severity was assessed with the Patient Health Questionnaire 8-item scale (PHQ-8), range 0–24, Cronbach’s alpha 0.82, with higher values indicating higher severity, score ≥10 corresponding to current depression [32]. Health-related quality of life (HR-QoL) was assessed with the Short-Form 12-item health survey (SF-12), range 0–100, Cronbach’s alpha 0.67; the EuroQol-5 Dimensions-5 levels (EQ-5D-5L), using the UK value sets, range 0–1, Cronbach’s alpha 0.73, with higher values indicating better health utility [33,34]; and EQ-VAS, range 0–100, with higher values indicating better HR-QoL. 

### 2.5. Other Measures

Sociodemographic characteristics (age, sex, household composition, educational level, income, migration background), BMI (body mass index), and the presence of chronic conditions were assessed by self-report questionnaires. Good adherence to interventions was defined a priori as attending ≥4 of 7 group sessions [35]. The satisfaction with the SEFAC intervention was evaluated with a questionnaire consisting of eight items at 6-month follow-up. Seven items were rated on a 5-point scale, ranging from strongly agree to strongly disagree. One item was rated on a 1-to-10 rating scale, with higher values indicating higher satisfaction. 

### 2.6. Power Considerations

The power considerations of the study were described previously [15]. It was planned to include 452 participants at baseline. By assuming a loss to follow-up of 20%, it was expected to have data of 360 participants with a baseline and a follow-up measure. Assuming equal standard deviations (SD) at baseline and follow-up, an alpha of 0.05 and power of 0.80, and by taking into account an average cluster size of 90 participants (360/4) and an intra-class correlation coefficient of 0.02, a difference of 0.24 SD between baseline and follow-up can be established for continuous outcome measures, such as the SF-12 [15].

### 2.7. Statistical Methods

Participant characteristics were described using mean (SD) or number of participants (%) for the total study sample. The effects of the intervention were assessed in the participants who completed the baseline and follow-up questionnaires and attended ≥4 sessions. A paired samples t-test was performed to assess the effects of the intervention on continuous outcome measures. Cohen’s *d* within-group effect size was computed for all significant outcomes [36]. For dichotomous outcome measures, the paired McNemar test was used. We stratified the descriptive statistics (see Supplement Table S2) and effects of the intervention on the outcome measures for the subgroups ‘History of CVD’ and ‘At risk of CVD’ (see Supplement Table S3a,b). The mean change in outcome measures for both subgroups was compared using an independent samples *t*-test for continuous outcomes and a *z*-test for dichotomous outcomes (see Supplement Table S3c). We did not impute missing data as only <5% of the data per variable were missing. Analyses were conducted with SPSS version 25.0 (IBM SPSS Statistics for Windows, IBM Corp., Armonk, NY, USA). The 2-sided significance threshold, after Bonferroni correction for multiple testing, was set at *p* = 0.05/20 = 0.0025. 

## 3. Results

### 3.1. Description of Participants

Between 28 November 2018 and 20 February 2020, 352 participants who fulfilled the inclusion criteria provided informed consent and started the intervention; 77 (21.9%) with a history of CVD and 275 (78.1%) at increased risk of CVD due to the presence of one or more risk factors. Participants were included in 3 European study sites: Rijeka, Croatia (*n* = 147); Treviso, Italy (*n* = 97); Rotterdam, the Netherlands (*n* = 84). At the pilot site in the UK (*n* = 91), outcome measurements with the self-report questionnaires were not feasible because of a high prevalence of health literacy problems; hence, these participants were not included for further analyses. Table 1 shows the baseline characteristics of the study sample. 

### 3.2. Adherence to Interventions and Follow-Up Attrition

**Figure 1:**
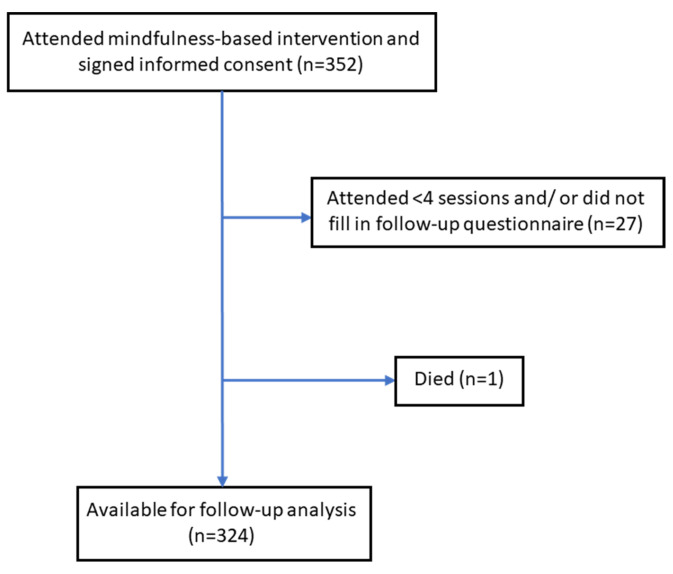
Participant flow chart.

Of all included participants (*n* = 352), 343 (97.4%) attended four or more of the seven group sessions. A median of 6 out of 7 group sessions (interquartile range [IQR], 6–7) were attended. The number of participants taking part in the 6-month follow-up assessment was 327 (92.9%). Overall, 324/352 participants (92.0%) completed the baseline and 6-month follow-up questionnaires, attended ≥4 sessions, and were included as the study sample for further analyses. Baseline characteristics of the 28 dropout participants were not significantly different from the included participants, except for the study site. The participant flow chart is shown in Figure 1.

### 3.3. Outcomes

Table 2 presents the outcome scores at baseline and follow-up of the 324 participants who completed both the baseline and 6-month follow-up questionnaires and who attended ≥4 sessions. Three of the four self-efficacy outcomes significantly improved, except for physical exercise self-efficacy. At the 6-month follow-up, there was a significant reduction in perceived stress and PHQ depression scores and significantly more social support as compared to baseline. Participants of the SEFAC intervention also experienced significant improvements in self-rated overall health and health utility (all *p* < 0.0025). Over time, no significant changes were observed for nutrition, physical activity, substance use, sleep, medication adherence, or mental and physical HR-QoL. 

### 3.4. Satisfaction and Adverse Events

Of those completing the questions on satisfaction with the intervention at the 6-month follow-up, participants reported that the intervention improved self-awareness (84.8%) and stimulated them to work on a healthy lifestyle (85.4%). The average satisfaction score was 8.2 ± 1.6 on a scale from 1 (lowest) to 10 (highest). The majority of participants (75.0%) perceived the SEFAC app as supportive of a healthy lifestyle through mindfulness exercises. (See Supplement Table S4). One participant died during the 6-month follow-up; this event was judged as unrelated to the intervention.

## 4. Discussion

This multicenter study involving over 300 individuals with or at increased risk of CVD showed modest effects from a 7-week MBI on self-efficacy, stress, depressive symptoms, social support, and self-rated overall health. 

The SEFAC MBI was primarily designed to enhance self-management as an approach to improving health behavior and well-being for individuals with or at increased risk of CVD. Self-efficacy, defined as the belief that one has the ability to accomplish a specific task or reach a goal [37], is considered a key modifiable mediator of self-management skills in chronic disease. Individuals with high self-efficacy are more likely to believe they can master challenging problems and recover quickly from setbacks and disappointments. Therefore, it is hypothesized that interventions that enhance self-efficacy can improve patient self-management of chronic conditions [26]. The participants’ self-efficacy in our intervention increased, except for physical exercise self-efficacy. The mean changes in self-efficacy were comparable to those found in other self-management interventions with a similar follow-up duration and have been shown to be associated with better health outcomes [23]. Our findings align with a recent study showing that an 8-week mindfulness training facilitated self-management skills among primary care patients diagnosed with mental health conditions [38]. Though self-efficacy is considered essential for initiating and maintaining health behavior change, the improvements in self-efficacy in our participants did not translate into healthier lifestyle behaviors relevant to cardiovascular outcomes, such as nutrition, physical activity, smoking cessation, and sleep. 

Despite widespread awareness that behavior change is key to the prevention and treatment of CVD, it remains challenging to change behavior [39]. Theoretically, training the mind to hold each moment in awareness may be paramount to change habit patterns [40]. Based on the limited number of studies, there is no definitive agreement yet in the literature concerning the effects of mindfulness interventions alone on health behaviors. Mindfulness has been shown to promote favorable changes in selected behaviors related to psychiatric conditions, such as several substance-use disorders and eating disorders [13]. However, our study’s lack of effect on nutrition, physical activity and other lifestyle factors does not support the benefits of a 7-week MBI for health behavior change related to CVD. This effect may, in part, be related to the way clinicians, patients, and the research community incorrectly conceptualize contemplative practices as expedient interventions for health problems. Training the mind in awareness and understanding the subtleties of one’s existence take time and consistent practice. A 7-week MBI is probably inadequate to achieve a level of expertise needed to transform behavior that depends on mastery of complex biological, mental and emotional processes. However, the results of this study do support the inclusion of mindfulness training in the arsenal of lifestyle interventions to combat CVD together with evidence-based nutrition and physical activity interventions.

Participation in the SEFAC MBI promoted psychological well-being, including reducing perceived stress, less depressive symptoms and better self-rated overall health. Previous meta-analyses demonstrated comparable small-to-moderate beneficial effects for MBIs on stress, depression, anxiety, and quality of life in individuals with a range of chronic conditions [11,12]. Psychological well-being has been identified as a positive cardiovascular health asset [4]. It is associated with improved CVD outcomes, whereas adverse psychological factors, such as stress and depression, are associated with an increased risk of cardiovascular events [41,42]. Psychological well-being, including positive thoughts and feelings, purpose in life, and optimism, is linked with cardiovascular health via neurobiological processes, behavioral pathways, and psychosocial resources that protect health and buffer stress effects [3,5]. Several guidelines and scientific statements on CVD prevention and management recommend screening for psychosocial risk factors and considering tailored interventions to enhance the quality of life as well as life expectancy and to reduce cardiovascular events [6,43]. In line with this, the observed improvement in psychological health six months after our 7-week MBI may benefit cardiovascular health. 

Previous studies have indicated that social isolation is a risk factor for the poor prognosis of individuals with cardiovascular disease. Isolated and lonely persons have a 1.5-fold increased risk of myocardial infarction, stroke and death [44]. Most of this excess risk is attributable to conventional risk factors, such as obesity, smoking, low education and pre-existing chronic illness [44]. In our study, one-third of the participants lived alone, and social support significantly improved following the MBI. Mindfulness practices may effectively reduce social risk by addressing both the subjective perception of loneliness and objective social isolation. In addition to the social context of a group-based MBI, the development of mindfulness-specific attention monitoring and acceptance skills is associated with improved social functioning [45]. These findings support group-based MBIs to promote the prevention and treatment of CVD in individuals with poor social connections. 

One of the strengths of our study is that it was conducted in multiple European study sites with heterogeneous populations of middle-aged and older adults with representative cardiovascular risk profiles. The results suggest that the SEFAC MBI applies to a broad range of individuals with or at increased risk of chronic CVD. The statistical power of the study was considered sufficient; instead of 360 participants, data of 352 and 324 participants were analyzed. Because of the limited sample sizes per study site, we could not generalize about differences in the effects of the MBI among the different study sites. Only about 10% of participants were lost to follow-up, which is a low dropout rate compared to similar behavioral interventions. Dropped out participants were not different from those with complete follow-up in terms of baseline characteristics, making attrition bias less likely.

This study has several limitations. First, due to the lack of a control group, it cannot be ruled out that the observed changes are due to nonspecific effects unrelated to the intervention, including attention, expectations for improvement, social support and sense of community deriving from participation in a group-based intervention. Furthermore, although our MBI was based on and included the mindfulness exercises of the well-tested MBSR curriculum, mindfulness itself as a psychological construct was not assessed in this study. All outcome measures were self-reported, which may be less accurate than objectively measured outcomes. In addition, we cannot exclude the presence of a selection or social desirability bias. Our mindfulness-based intervention lasted seven weeks, unlike other existing programs that last eight weeks. However, the SEFAC mindfulness-based intervention took place in relatively small groups, with a similar duration as the MBSR and MBCT; therefore, the intensity of the intervention was assumed to be relatively high. Nevertheless, we recommend that future studies consider possibilities to strengthen the intervention, including making it last eight weeks. In addition, for future studies with larger varied samples, we advise exploring the differences regarding the effects between subgroup participants with cardiovascular disease and subgroup with participants at risk of cardiovascular disease. We recommend future studies to apply a randomized controlled trial (RCT) with pre-defined primary and secondary outcomes. This study only evaluated the ‘mid-term’ effects of the intervention after six months; we recommend future studies to measure both ‘immediate’ effects after finishing the intervention (i.e., after seven weeks in the current intervention), and the ‘mid-term’ effects after six months. In our study, three scales showed a Cronbach’s Alpha <0.70; we recommend that future studies pay attention to the reliability of the measurements. Moreover, an intervention such as the current one that is applied only one time might have been too short since changing lifestyle patterns is complex and requires continuous attention, practice, and support. Finally, the predefined 6-month follow-up period may have been too short to assess the durability of the observed effects. 

## 5. Conclusions

In conclusion, the present study shows promising effects of a mindfulness-based intervention, in combination with social engagement and e-health support. It enhances self-management as a complementary approach for modifying psychosocial factors relevant to cardiovascular care. Our findings deserve further study in a larger, long-term randomized controlled trial to explore the potential of mindfulness training in catalyzing chronic disease self-management. An intervention offering access to high-quality therapeutic lifestyle interventions focusing on nutrition, physical activity, and substance use, in conjunction with ongoing encouragement for mindfulness practice with digital and community resources, may be a sustainable approach for promoting cardiovascular health. 

## Figures and Tables

**Table 1:** Baseline characteristics of the SEFAC study sample (*n* = 352).

	Study Sample (*n* = 352)
Age, y	66.7 (7.9) *
Female sex	280 (79.5%)
Study site	
Croatia	149 (42.3%)
Italy	97 (27.6%)
The Netherlands	106 (30.1%)
History of CVD	77 (21.9%)
At risk of CVD ^#^	275 (78.1%)
T2DM	63 (17.9%)
Hypertension	173 (49.1%)
Hypercholesterolemia	173 (49.1%)
Other chronic conditions	
Cancer	56 (15.9%)
(Osteo)arthritis	140 (39.8%)
Pulmonary condition(s)	48 (13.6%)
Gastrointestinal condition(s)	24 (6.8%) *
Current smoking	34 (9.7%)
Alcohol use ≥ 4 times/wk	43 (12.2%)
Aerobic physical activity < 150 min/wk	145 (41.2%)
Fruit < 3 servings/d	312 (88.9%) *
Vegetables < 3 servings/d	325 (93.1%) †
Overweight (BMI ≥ 25 kg/m^2^)	226 (64.2%)
Current depression (PHQ-8 ≥ 10)	64 (18.2%)
Moderate-high perceived stress (PSS-10 ≥ 14)	245 (69.6%)
Living alone	123 (34.9%)
Education	
Primary or no education	54 (15.3%)
Secondary	164 (46.6%)
Tertiary or higher	134 (38.1%)
Low income (decile 1 and 2)	44 (13.0%) ‡
Migration background	52 (14.8%)

Data are mean (SD) or number of participants (%). Missing items: * *n* = 1; † *n* = 3; ‡ *n* = 14, ^#^ Some participants fell into more than 1 subcategory of ‘At risk of CVD’. Abbreviations: CVD, cardiovascular disease; T2DM, type 2 diabetes mellitus; BMI, body mass index; PHQ-8, Patient Health Questionnaire; PSS-10, Perceived Stress Scale; SD, standard deviation.

**Table 2:** Effects of SEFAC mindfulness-based intervention (*n* = 324).

Outcomes	Baseline	Follow-Up (6 Month)	Effect Variable	Estimate	Confidence Interval	*p*-Value ^#^
Self-efficacy						
SEMCD (range 1–10) ^§^	7.0 (1.6)	7.3 (1.7)	Mean change	0.322 ^a^	0.153–0.492	<0.001 *
GSES (range 10–40) ^§^	30.5 (5.4)	31.9 (5.3)	Mean change	1.420 ^b^	0.881–1.958	<0.001 *
PESES (range 5–20) ^§^	13.5 (3.9)	14.1 (4.1)	Mean change	0.534	0.084–0.984	0.020 *
NSES (range 5–20) ^§,1^	13.9 (3.7)	14.7 (3.7)	Mean change	0.771 ^c^	0.391–1.151	0.000 *
Health behaviors						
Nutrition						
Fruit ≥ 3 portions/d ^1^	36 (11.1%)	39 (12.1%)	OR	1.15	0.63–2.09	0.761 ^†^
Vegetables, ≥3 portions/d ^2^	22 (6.8%)	26 (8.1%)	OR	1.27	0.64–2.46	0.607 ^†^
Physical activity						
Stretching/strengthening (min/wk) ^3^	55.5 (61.9)	50.3 (61.7)	Mean change	−5.203	−12.452–2.046	0.159 *
Aerobic exercise (min/wk)	164.0 (100.1)	176.8 (112.9)	Mean change	12.824	1.403–24.245	0.028 *
Sedentary behavior (h/d) ^2^	5.8 (2.5)	5.4 (2.6)	Mean change	−0.390	−0.649–−0.130	0.003 *
Substance use						
Current smoking	33 (10.2%)	29 (9.0%)	OR	0.20	0.02–1.71	0.219 ^†^
Alcohol, 4 times/wk or more	40 (12.3%)	35 (10.8%)	OR	0.67	0.30–1.48	0.424 ^†^
Stress management						
Perceived stress (PSS-10; range 0–40) ^$^	16.3 (5.9)	15.1 (5.7)	Mean change	−1.216 ^d^	−1.759–−0.673	<0.001 *
Sleep						
Sleep problems (range 1–10) ^$^	4.7 (2.6)	4.3 (2.6)	Mean change	−0.417	−0.698–−0.136	0.004 *
Fatigue (range 1–10) ^$^	4.8 (2.3)	4.7 (2.4)	Mean change	−0.093	−0.356–0.171	0.490 *
Relationships						
Social support (OSSS-3; range 3–14) ^§,1^	9.4 (2.2)	9.7 (2.3)	Mean change	0.334 ^e^	0.121–0.548	0.002 *
Medication adherence						
SMAQ (no adherence) ^4^	170 (59.9%)	154 (54.2%)	OR	0.64	0.03–1.03	0.081 ^†^
Depression						
PHQ-8 (range 0–24) ^$^	5.7 (4.2)	5.0 (4.0)	Mean change	−0.741 ^f^	−1.093–−0.388	<0.001 *
HR-QoL						
PCS (SF-12; range 0–100) ^§,2^	44.6 (9.1)	45.8 (8.9)	Mean change	1.156	0.344–1.968	0.005 *
MCS (SF-12; range 0–100) ^§,2^	45.4 (10.1)	46.6 (9.2)	Mean change	1.148	0.161–2.136	0.023 *
EQ-5D-5L utility values (range <0–1) ^§,1^	0.80 (0.15)	0.82 (0.16)	Mean change	0.025 ^g^	0.010–0.039	<0.001 *
EQ-5D-5L overall health (range 0–100) ^§^	70.9 (16.6)	73.9 (17.5)	Mean change	3.022 ^h^	1.297–4.746	<0.001 *

Data shown are the available data of the 324 participants who completed the baseline and follow-up questionnaires and attended ≥4 of 7 SEFAC sessions. Data are mean (SD) or number of participants (%). The effect variable shows ‘mean change’ for continuous variables or ‘odds ratio’ for dichotomous variables. Missing items: ^1^ *n* = 1; ^2^ *n* = 2; ^3^ *n* = 4; ^4^ *n* = 40. Cohen’s *d* effect size: ^a^ *d* = 0.20; ^b^ *d* = 0.26; ^c^ *d* = 0.22; ^d^ *d* = 0.21; ^e^ *d* = 0.15; ^f^ *d* = 0.18; ^g^ *d* = 0.15; ^h^ *d* = 0.18. Abbreviations: SEFAC, Social Engagement Framework for Addressing the Chronic-disease-challenge; SEMCD, Self-Efficacy for Managing Chronic Disease scale; GSES, General Self-Efficacy Scale; PESES, Physical Exercise Self-Efficacy Scale; NSES, Nutrition Self-Efficacy Scale; OR, odds ratio; PSS-10, Perceived Stress Scale; OSSS-3, Oslo Social Support Scale; SMAQ, Short Medication Adherence Questionnaire; PHQ-8, Patient Health Questionnaire; HR-QoL, Health-related quality of life; PCS, Physical Component Summary of the SF-12; MCS, Mental Component Summary of the SF-12; SF-12, Short Form health survey; EQ-5D-5L, EuroQol-5 Dimensions-5 level. * *p*-value based on paired *t*-test; significant *p*-values in bold. ^†^ *p*-value based on McNemar test; significant *p*-values in bold. ^$^ A lower score is better. ^§^ A higher score is better. ^#^ Significant *p*-values in bold after Bonferroni correction for multiple testing was applied (*p* = 0.05/20 = 0.0025).

## Data Availability

The data presented in this study are not publicly available due to privacy reasons.

## Supplementary Materials

The following supporting information can be downloaded at: https://www.mdpi.com/article/10.3390/ijerph192013145/s1, Supplement Table S1. Overview SEFAC intervention; Supplement Table S2. Baseline characteristics of the SEFAC study sample, stratified by subgroups ‘History of CVD’ and ‘At risk of CVD’ (*n* = 352); Supplement Table S3a. Effects of the SEFAC intervention, stratified by subgroups ‘History of CVD’ and ‘At risk of CVD’, showing subgroup ‘History of CVD’ (*n* = 69); Supplement Table S3b. Effects of the SEFAC intervention, stratified by subgroups ‘History of CVD’ and ‘At risk of CVD’, showing subgroup ‘At risk of CVD’ (*n* = 255); Supplement Table S3c. Comparing mean change in outcome measures of the SEFAC intervention in subgroups ‘History of CVD (*n* = 69)’ and ‘At risk of CVD (*n* = 255)’; Supplement Table S4. Satisfaction with the SEFAC intervention at follow-up (*n* = 324). Supplement Figure S1a. Baseline questionnaire; Supplement Figure S1b. Follow-up questionnaire.

## Non-Standard Abbreviations and Acronyms

AUDIT-C	Alcohol Use Disorder Identification Test-Consucmption
BMI	Body Mass Index
CVD	cardiovascular disease
EQ-5D-5L	EuroQol-5 Dimensions-5
EQ-VAS	EuroQol visual analogue score
GSES	General Self-efficacy Scale
HR-QoL	health-related quality of life
IPAQ	International Physical Activity Questionnaire
MBI	mindfulness-based intervention
MBSR	mindfulness-based stress reduction
NSES	Nutrition Self-Efficacy Scale
OSSS-3	Oslo Social Support 3-item scale
PHQ-8	Patient Health Questionnaire 8-item scale
PSES	Physical Exercise Self-Efficacy Scale
PSS-10	Perceived Stress 10-item
SEFAC	the Social Engagement Framework for Addressing the Chronic-disease-challenge
SEMCD-6	Self-Efficacy for Managing Chronic Diseases 6-item scale
SD	standard deviation
SF-12	Short-Form 12-item health survey
SMAQ	Simplified Medication Adherence Questionnaire
T2DM	type 2 diabetes
VAS	visual analogue scale
