# Supplementary material for: Evaluation of an Intervention to Promote Self-Management Regarding Cardiovascular Disease: The Social Engagement Framework for Addressing the Chronic-Disease-Challenge (SEFAC)
Source: Int J Environ Res Public Health. 2022 Oct 12;19(20):13145. doi: 10.3390/ijerph192013145 (PMC9603702; doi:10.3390/ijerph192013145)
Supplement: Supplementary file 1 [file ijerph-19-13145-s001.zip › Supplement Figure 1a - Baseline questionnaire.pdf]

SEFAC

Social Engagement Framework for Addressing the Chronic disease challenge

INFORMED CONSENT

- I have read the patient information letter. I was able to ask additional questions. My questions have been answered. I have had enough time to decide if I want to participate.
- I understand that participation is completely voluntary. I understand that I can decide to discontinue participation at any time.
- I do not have to give a reason in case I decide to discontinue my participation in this study.
- I know some people can see my data. Those people are listed in the patient information letter. I give permission to use my data for the purposes listed in the patient information letter.
- I give permission to store my data for a maximum of 15 years after termination of this research.
- I agree to participate in this research.

Name and surname	
Date of birth	
Address	
Postcode and city	
Email address	
Telephone number	

Date ____________________ Signature ______________________________

BASELINE MEASUREMENT

SEFACquestUKv2.indd 1 15/11/2018 18:35:49

Country code

Participant number

INSTRUCTIONS FOR FILLING IN THE QUESTIONNAIRE

- Please answer all the questions, even if they seem to be the same: these questions help us to view the situation again from a different angle.
- Please tick one answer per question. If it is possible to tick more than one answer, this will be mentioned for this specific question.
- Please answer the questionnaire with a blue or black pen.
- When you are done, please check that you have not forgotten any questions.

Making a mistake
If you select a wrong box and want to correct, colour the wrong box black.

Example: You are female

1 What is your gender?

☒ Male
☐ Female

You select a wrong box and want to correct

1 What is your gender?

■ Male
☒ Female

Corrected: you have now answered that you are female

Text or numbers should be filled in within the box.

2 How many cups of coffee did you drink yesterday?

2 Correct

2 Wrong

SEFACquestUKv2.indd 2 15/11/2018 18:35:49

A • YOUR HEALTH AND WELL-BEING

This survey asks for your views about your health. This information will help keep track of how you feel and how well you are able to do your usual activities. Answer each question by choosing just one answer. If you are unsure how to answer a question, please give the best answer you can.

A1 In general, would you say your health is

- ☐ Excellent
- ☐ Very good
- ☐ Good
- ☐ Fair
- ☐ Poor

The following questions are about activities you might do during a typical day. Does *your health now* limit you in these activities? If so, how much?

	Yes, limited a lot	Yes, limited a little	No, not limited at all
**A2** Moderate activities such as moving a table, pushing a vacuum cleaner, swimming or cycling	☐	☐	☐
**A3** Climbing several flights of stairs	☐	☐	☐

During the *past 4 weeks*, have you had any of the following problems with your work or other regular daily activities *as a result of your physical health?*

	All of the time	Most of the time	Some of the time	A little of the time	None of the time
**A4** Accomplished less than you would like	☐	☐	☐	☐	☐
**A5** Were limited in the kind of work or other activities	☐	☐	☐	☐	☐

During the *past 4 weeks*, have you had any of the following problems with your work or other regular daily activities *as a result of any emotional problems* (such as feeling depressed or anxious)?

	All of the time	Most of the time	Some of the time	A little of the time	None of the time
**A6** Accomplished less than you would like	☐	☐	☐	☐	☐
**A7** Were limited in the kind of work or other activities	☐	☐	☐	☐	☐

A8 During the *past 4 weeks*, how much did *pain* interfere with your normal work (including work out-side the home and household chores)?

- ☐ Not at all
- ☐ A little bit
- ☐ Moderately
- ☐ Quite a bit
- ☐ Extremely

SEFACquestUKv2.indd 3 15/11/2018 18:35:49

These questions are about how you have been feeling during the *past 4 weeks*. For each question, please give the one answer that comes closest to the way you have been feeling.

**How much of the time during the *past 4 weeks…***	All of the time	Most of the time	Some of the time	A little of the time	None of the time
**A9** Have you felt calm and peaceful?	☐	☐	☐	☐	☐
**A10** Did you have a lot of energy?	☐	☐	☐	☐	☐
**A11** Have you felt down-hearted and blue?	☐	☐	☐	☐	☐

A12 During the *past 4 weeks*, how much have your *physical health or emotional problems* interfered with your normal social activities with family, friends, neighbours, or groups?

- ☐ All of the time
- ☐ Most of the time
- ☐ Some of the time
- ☐ A little of the time
- ☐ None of the time

B • YOUR QUALITY OF LIFE

B1 We are interested in learning whether or not you are affected by *sleep problems.* Please check the box below that describes your sleep in the *past week*.

No problem sleeping	☐ 1	☐ 2	☐ 3	☐ 4	☐ 5	☐ 6	☐ 7	☐ 8	☐ 9	☐ 10	Very big problem sleeping

B2 We are interested in learning whether or not you are affected by *fatigue.* Please check the box below that describes your fatigue in the *past week*.

No fatigue	☐ 1	☐ 2	☐ 3	☐ 4	☐ 5	☐ 6	☐ 7	☐ 8	☐ 9	☐ 10	Severe fatigue

B3 Please tick the one box that best describes your health *today*.

- ☐ I have no problems walking about
- ☐ I have slight problems walking about
- ☐ I have moderate problems walking about
- ☐ I have severe problems walking about
- ☐ I am unable to walk about

B4 Please tick the one box that best describes your health *today*.

- ☐ I have no problems washing or dressing myself
- ☐ I have slight problems washing or dressing myself
- ☐ I have moderate problems washing or dressing myself
- ☐ I have severe problems washing or dressing myself
- ☐ I am unable to wash or dress myself

B5 Please tick the one box that best describes your health *today*.

- ☐ I have no problems doing my usual activities
- ☐ I have slight problems doing my usual activities
- ☐ I have moderate problems doing my usual activities
- ☐ I have severe problems doing my usual activities
- ☐ I am unable to do my usual activities

B6 Please tick the one box that best describes your health *today*.

- ☐ I have no pain or discomfort
- ☐ I have slight pain or discomfort
- ☐ I have moderate pain or discomfort
- ☐ I have severe pain or discomfort
- ☐ I have extreme pain or discomfort

B7 Please tick the one box that best describes your health *today*.

- ☐ I am not anxious or depressed
- ☐ I am slightly anxious or depressed
- ☐ I am moderately anxious or depressed
- ☐ I am severely anxious or depressed
- ☐ I am extremely anxious or depressed

B8 We would like to know how good or bad your health is *today*.

- This scale is numbered from 0 to 100.
- 100 means the *best* health you can imagine.
- 0 means the *worst* health you can imagine.
- Mark an X on the scale to indicate how your health is <u>today</u>.
- Now, please write the number you marked on the scale in the box below.

Your health <u>today</u>:

☐☐☐

The *best* health
you can imagine

100
95
90
85
80
75
70
65
60
55
50
45
40
35
30
25
20
15
10
5
0

The *worst* health
you can imagine

C • YOUR LIFESTYLE

C1 How *often* do you eat breakfast?

- ☐ 0 days per week
- ☐ 1 day per week
- ☐ 2 days per week
- ☐ 3 days per week
- ☐ 4 days per week
- ☐ 5 days per week
- ☐ 6 days per week
- ☐ 7 days per week

C2 How *many* servings of 'fresh' vegetables do you usually eat? *One serving corresponds to one serving spoon (50–100 grams). Examples of vegetables are: green beans, carrots, broccoli, sprouts, tomatoes, salad, spinach, leek or cucumber.*

- ☐ Less than 1 per week
- ☐ 1-2 per week
- ☐ 3-4 per week
- ☐ 5-6 per week
- ☐ 1-2 per day
- ☐ 3 or more per day

SEFACquestUKv2.indd 6 15/11/2018 18:35:49

C3 How *many* servings of 'fresh' fruit do you usually eat? *One serving corresponds to one handful. Examples of servings of fruit are: an apple, a pear, a kiwi, a banana, an orange, a handful of strawberries, a handful of berries or a handful of grapes.*

- ☐ Less than 1 per week
- ☐ 1-2 per week
- ☐ 3-4 per week
- ☐ 5-6 per week
- ☐ 1-2 per day
- ☐ 3 or more per day

C4 How *often* do you have a drink containing *alcohol?*

- ☐ Never
- ☐ Monthly or less
- ☐ 2-4 times a month
- ☐ 2-3 times a week
- ☐ 4 or more times a week

C5 Do you smoke at the present time?

- ☐ Yes
- ☐ No

C6 During the past week, even if it was not a typical week for you, how much *total* time (for the *entire week)* did you spend on each of the following?

	None	Less than 30 minutes a week	30-60 minutes a week	1-3 hours a week	More than 3 hours a week
a. Stretching or strengthening exercises (range of motion, weights, etc.)	☐	☐	☐	☐	☐
b. Walking	☐	☐	☐	☐	☐
c. Swimming or aquatic exercise	☐	☐	☐	☐	☐
d. Bicycling (including stationary exercise bikes)	☐	☐	☐	☐	☐
e. Other aerobic exercise equipment (stairmaster, rowing, treadmill etc.)	☐	☐	☐	☐	☐
f. Other aerobic exercise (specify:)	☐	☐	☐	☐	☐

C7 The next question is about the time you spent *sitting* during the *past week*. *Include time spent at work, at home, while doing course work and during leisure time. This may include time spent sitting at a desk, visiting friends, reading, or sitting or lying down to watch television.*

	Number of hours per day
a. During the *past week,* how much time did you spend *sitting* on a *week day?*	☐☐
b. During the *past week,* how much time did you spend *sitting* on a *weekend day?*	☐☐

SEFACquestUKv2.indd 7 15/11/2018 18:35:49

D • YOUR MOOD

These questions ask you about your feelings and thoughts *during the last month*. In each case, you will be asked to indicate how often you felt or thought a certain way.

		Never	Almost never	Some-times	Fairly often	Very often
**D1**	In the last month, how often have you been upset because of something that happened unexpectedly?	☐	☐	☐	☐	☐
**D2**	In the last month, how often have you felt that you were unable to control the important things in your life?	☐	☐	☐	☐	☐
**D3**	In the last month, how often have you felt nervous and “stressed”?	☐	☐	☐	☐	☐
**D4**	In the last month, how often have you felt confident about your ability to handle your personal problems?	☐	☐	☐	☐	☐
**D5**	In the last month, how often have you felt that things were going your way?	☐	☐	☐	☐	☐
**D6**	In the last month, how often have you found that you could not cope with all the things that you had to do?	☐	☐	☐	☐	☐
**D7**	In the last month, how often have you been able to control irritations in your life?	☐	☐	☐	☐	☐
**D8**	In the last month, how often have you felt that you were on top of things?	☐	☐	☐	☐	☐
**D9**	In the last month, how often have you been angered because of things that happened that were outside of your control?	☐	☐	☐	☐	☐
**D10**	In the last month, how often have you felt difficulties were piling up so high that you could not overcome them?	☐	☐	☐	☐	☐

D11 Over the *last 2 weeks*, how often have you been bothered by any of the following problems?

	Not at all	Several days	More than half of the days	Nearly every day
a. Little interest or pleasure in doing things.	☐	☐	☐	☐
b. Feeling down, depressed, or hopeless.	☐	☐	☐	☐
c. Trouble falling or staying asleep, or sleeping too much.	☐	☐	☐	☐
d. Feeling tired or having little energy.	☐	☐	☐	☐
e. Poor appetite or overeating.	☐	☐	☐	☐
f. Feeling bad about yourself, or that you are a failure, or have let yourself or your family down.	☐	☐	☐	☐
g. Trouble concentrating on things, such as reading the newspaper or watching television.	☐	☐	☐	☐
h. Moving or speaking so slowly that other people could have noticed. Or the opposite – being so fidgety or restless that you have been moving around a lot more than usual.	☐	☐	☐	☐

SEFACquestUKv2.indd 8 15/11/2018 18:35:50

E • YOUR RESILIENCE

**Please tick the one box that best describes your *current* beliefs.**	Not at all true	Hardly true	Moderately true	Exactly true
**E1** I can always manage to solve difficult problems if I try hard enough.	☐	☐	☐	☐
**E2** If someone opposes me, I can find the means and ways to get what I want.	☐	☐	☐	☐
**E3** It is easy for me to stick to my aims and accomplish my goals.	☐	☐	☐	☐
**E4** I am confident that I could deal efficiently with unexpected events.	☐	☐	☐	☐
**E5** Thanks to my resourcefulness, I know how to handle unforeseen situations.	☐	☐	☐	☐
**E6** I can solve most problems if I invest the necessary effort.	☐	☐	☐	☐
**E7** I can remain calm when facing difficulties because I can rely on my coping abilities.	☐	☐	☐	☐
**E8** When I am confronted with a problem, I can usually find several solutions.	☐	☐	☐	☐
**E9** If I am in trouble, I can usually think of a solution.	☐	☐	☐	☐
**E10** I can usually handle whatever comes my way.	☐	☐	☐	☐

E11 Please rate each of the following statements.

a. How sure are you that you can keep the *fatigue* caused by your disease from interfering with the things you want to do?

Not sure at all	1	2	3	4	5	6	7	8	9	10	Totally sure
	☐	☐	☐	☐	☐	☐	☐	☐	☐	☐	

b. How sure are you that you can keep the *physical discomfort or pain* of your disease from interfering with the things you want to do?

Not sure at all	1	2	3	4	5	6	7	8	9	10	Totally sure
	☐	☐	☐	☐	☐	☐	☐	☐	☐	☐	

c. How sure are you that you can keep the *emotional distress* caused by your disease from interfering with the things you want to do?

Not sure at all	1	2	3	4	5	6	7	8	9	10	Totally sure
	☐	☐	☐	☐	☐	☐	☐	☐	☐	☐	

d. How sure are you that you can keep any *other symptoms or health problems* you have from interfering with the things you want to do?

Not sure at all	1	2	3	4	5	6	7	8	9	10	Totally sure
	☐	☐	☐	☐	☐	☐	☐	☐	☐	☐	

e. How sure are you that you can do the different tasks and activities needed to manage your health condition so as to reduce your need to see a doctor?

Not sure at all	1	2	3	4	5	6	7	8	9	10	Totally sure
	☐	☐	☐	☐	☐	☐	☐	☐	☐	☐	

f. How sure are you that you can do things other than just taking medication to reduce how much your illness affects your everyday life?

Not sure at all	1 ☐	2 ☐	3 ☐	4 ☐	5 ☐	6 ☐	7 ☐	8 ☐	9 ☐	10 ☐	Totally sure

How certain are you that you could overcome the following barriers?

I can manage to carry out my exercise intentions …	Very uncertain	Rather uncertain	Rather certain	Very certain
**E12** ... even when I have worries and problems	☐	☐	☐	☐
**E13** ... even if I feel depressed	☐	☐	☐	☐
**E14** ... even when I feel tense	☐	☐	☐	☐
**E15** ... even when I am tired	☐	☐	☐	☐
**E16** ... even when I am busy	☐	☐	☐	☐

How certain are you that you could overcome the following barriers?

I can manage to stick to healthful foods …	Very uncertain	Rather uncertain	Rather certain	Very certain
**E17** ... even if I need a long time to develop the necessary routines	☐	☐	☐	☐
**E18** ... even if I have to try several times until it works	☐	☐	☐	☐
**E19** ... even if I have to rethink my entire way of nutrition	☐	☐	☐	☐
**E20** ... even if I do not receive a great deal of support from others when making my first attempts	☐	☐	☐	☐
**E21** ... even if I have to make a detailed plan	☐	☐	☐	☐

F • YOUR MEDICATION USE *(Please skip to question G1 if you don't take any medicines.)*

The following questions refer to any medication you regularly take.

F1 Do you always take your medication at the specified time?

- ☐ Yes
- ☐ No

F2 When you feel ill, have you ever discontinued taking your medication?

- ☐ Yes
- ☐ No

F3 Have you ever forgotten to take your medication?

- ☐ Yes
- ☐ No → *Please skip to **question F5***

SEFACquestUKv2.indd 10 15/11/2018 18:35:50

F4 Have you ever forgotten to take your medication during the *weekend*?

- ☐ Yes
- ☐ No

F5 In the *last week*, how many times did you fail to take your prescribed dose?

- ☐ Never
- ☐ 1 - 2 times
- ☐ 3 - 5 times
- ☐ 6 - 10 times
- ☐ More than 10 times

F6 Since the last medical visit, how many whole days have gone by in which you did not take your medication?

☐☐☐ days

G • THE CARE YOU RECEIVE

The following questions refer to the care you received for any reason (not specifically your chronic condition).

G1 In the *past 6 months*, how many times did you visit a medical doctor (general practitioner or specialist, at a doctor's practice or hospital's outpatient department)? *Do not include visits while in the hospital or to a hospital's Accident and Emergency.*

- ☐ Never
- ☐☐ times

G2 In the *past 6 months*, how many times did you go to a hospital's Accident and Emergency?

- ☐ Never
- ☐☐ times

G3 How many different times did you stay in a hospital overnight or longer in the *past 6 months*?

- ☐ Never
- ☐☐ times

SEFACquestUKv2.indd 11 15/11/2018 18:35:50

G4 How many total nights did you spend in the hospital in the *past 6 months?*

☐ Never

☐☐ nights in total in the past 6 months

For *unpaid work,* you can be bothered by physical or psychological problems. Sometimes as a result you (might) do less. For example you have trouble caring for your children or doing voluntary work. Or you are unable to run errands and pick up groceries, or to work in the garden. The following questions refer to this.

G5 In the *past 4 weeks*, were there days in which you were forced to do less unpaid work because of physical or psychological problems?

☐ No → *Please skip to* ***question G8*** *of this questionnaire.*

☐ Yes

G6 In the *past 4 weeks*, on how many days did this happen?

☐☐ days

G7 Imagine that somebody, for example your partner, family member or friend helped you on these days, and he or she did all the *unpaid work* that you were unable to do for you. How many hours on average did that person spend doing this on these days?

☐☐ hours

G8 How many people are so close to you that you can count on them if you have serious problems?

☐ None

☐ 1 - 2

☐ 3 - 5

☐ 6 or more

G9 How much concern do people show in what you are doing?

☐ A lot of concern and interest

☐ Some concern and interest

☐ Uncertain

☐ Little concern and interest

☐ No concern and interest

G10 How easy can you get practical help from neighbors if you should need it?

- ☐ Very easy
- ☐ Easy
- ☐ Possible
- ☐ Difficult
- ☐ Very difficult

G11 Due to your health, did you receive help in household work in the *past 4 weeks*? *(Examples are cleaning, cooking, grocery shopping by a community nurse or home help.)*

- ☐ No
- ☐ Yes, ☐☐ hours per week, during ☐ weeks

G12 Did you receive help in caring for yourself in the *past 4 weeks*? *(Examples are help dressing or washing by a community nurse or home help.)*

- ☐ No
- ☐ Yes, ☐☐ hours per week, during ☐ weeks

H • YOUR PERSONAL DETAILS

H1 Today's date is:

☐☐ – ☐☐ – 20 ☐☐

day month year

H2 Which sex are you?

- ☐ Male ☐ Female
- ☐ Other *(please specify:)*

H3 What is your age? ☐☐☐ years

H4 How tall are you? ☐☐☐ cm

H5 Approximately how much do you weigh? ☐☐☐ kg

H6 Has a doctor ever told you that you had one or more of these conditions?

	Yes	No
a. A heart attack including myocardial infarction or coronary thrombosis or any other heart problem including congestive heart failure	☐	☐
b. High blood pressure or hypertension	☐	☐
c. High blood cholesterol	☐	☐
d. A stroke or cerebral vascular disease	☐	☐
e. Diabetes or high blood sugar	☐	☐
f. Chronic lung disease such as chronic bronchitis, emphysema or COPD	☐	☐
g. Asthma	☐	☐
h. Arthritis, including osteoarthritis or rheumatism	☐	☐
i. Osteoporosis	☐	☐
j. Cancer or malignant tumour, including leukaemia or lymphoma, but excluding minor skin cancers	☐	☐
k. Stomach or duodenal ulcer, peptic ulcer	☐	☐
l. Parkinson's disease	☐	☐
m. Cataract	☐	☐
n. Hip fracture or femoral fracture	☐	☐
o. Other chronic condition, namely …	☐	☐

H7 In which country were you born?

☐ The United Kingdom

☐ Other, namely:

H8 In which country was your mother born?

☐ The United Kingdom

☐ Other, namely:

H9 In which country was your father born?

☐ The United Kingdom

☐ Other, namely:

SEFACquestUKv2.indd 14 15/11/2018 18:35:50

Country code | Participant number

H10 What is your marital status?

- ☐ Single
- ☐ Married
- ☐ Long-term cohabitation, unmarried
- ☐ Widowed

H11 How many persons are living in your household?

☐☐ persons

H12 What is your household composition?

- ☐ Living alone
- ☐ Living with partner, no children
- ☐ Living with partner and children
- ☐ Living without partner, with children
- ☐ Living in a household shared with others

H13 What is the highest level of education you have completed? *(Check one box only.)*

- ☐ No education
- ☐ Primary education
- ☐ Lower or upper secondary education
- ☐ Post-secondary non-tertiary education or short-cycle tertiary education
- ☐ Bachelor or equivalent
- ☐ Master or equivalent
- ☐ Doctoral or equivalent

H14 Which category indicates your net *__monthly__* household income?

- ☐ Less than £ 713
- ☐ £ 713 – £ 955
- ☐ £ 956 – £ 1194
- ☐ £ 1195 – £ 1446
- ☐ £ 1447 – £ 1759
- ☐ £ 1760 – £ 2137
- ☐ £ 2138 – £ 2571
- ☐ £ 2572 – £ 3171
- ☐ £ 3172 – £ 4175
- ☐ £ 4176 or more

H15 Below are various possible sources of income. Which kinds of income did your household receive in the *__past year__*? *(Please select all boxes that apply.)*

- ☐ Income from work (as employee or self-employed)
- ☐ Old age pension
- ☐ Family/children related allowances
- ☐ Social security benefits
- ☐ Other sources of income not mentioned above
- ☐ No source of income

H16 What is your occupation?

H17 How many hours a week do you work? *(Count only the hours that you get paid.)*

☐☐ hours

H18 How many days a week do you work?

☐ days

H19 Have you missed work in the *last 4 weeks* as a result of being sick? *(Only count the missed work days in the last 4 weeks.)*

- ☐ No → *Please skip to **question I1** of this questionnaire.*
- ☐ Yes, I have missed...

  ☐☐ days

H20 Did you miss work earlier than the *period of 4 weeks* due to being sick? *This is referring to one whole uninterrupted period of missed work as a result of being sick.*

- ☐ No → *Please skip to **question I1** of this questionnaire.*
- ☐ Yes

H21 How many days did the whole uninterrupted period of missed work last?

☐☐☐ days

I • ADDITIONAL REMARKS

I1 You will receive a questionnaire again in 6 months. How would you prefer to receive it?

- ☐ No preference
- ☐ Via email. *You will receive an email with a link to fill out the questionnaire online. Please make sure your email address is correctly stated on the front page.*
- ☐ Via mail. *You will receive a paper version of the questionnaire, which you will be able to return to us by mail. Please make sure your mail address is correctly stated on the front page.*

I2 Do you have any additional remarks that have not been addressed in this survey?

Thank you for completing this questionnaire!
