# Supplementary material for: Evaluation of an Intervention to Promote Self-Management Regarding Cardiovascular Disease: The Social Engagement Framework for Addressing the Chronic-Disease-Challenge (SEFAC)
Source: Int J Environ Res Public Health. 2022 Oct 12;19(20):13145. doi: 10.3390/ijerph192013145 (PMC9603702; doi:10.3390/ijerph192013145)
Supplement: Supplementary file 1 [file ijerph-19-13145-s001.zip › Supplementary Table S3b_final.pdf]

Supplementary Table 3b. Effects of the SEFAC intervention, stratified by subgroups 'History of CVD' and 'At risk of CVD', showing subgroup 'At risk of CVD' (n=255)

Outcomes	Baseline	Follow-up (6 month)	Effect variable	Estimate	Confidence interval	P-value#
**Self-efficacy**						
SEMCD (range 1-10) §	7.0 (1.6)	7.3 (1.7)	Mean change	0.309	0.124 – 0.494	**0.001***
GSES (range 10-40) §	30.6 (5.1)	31.9 (5.2)	Mean change	1.262	0.679 – 1.846	**<0.001***
PESES (range 5-20) §	13.6 (4.0)	14.1 (4.2)	Mean change	0.537	0.027 – 1.047	0.039*
NSES (range 5-20) §	13.8 (3.8)	14.5 (3.8)	Mean change	0.710	0.277 – 1.142	**0.001***
**Health behaviors**						
Nutrition						
Fruit ≥3 portions/d	24 (9.2%)	28 (11.0%)	OR	1.29	0.64 – 2.59	0.596†
Vegetables, ≥3 portions/d	15 (5.9%)	18 (7.1%)	OR	1.30	0.57 – 2.96	0.678†
Physical activity						
Stretching/strengthening (min/wk)	58.4 (62.9)	53.0 (62.5)	Mean change	-5.374	-13.498 – 2.750	0.194*
Aerobic exercise (min/wk)	158.6 (98.1)	175.8 (114.8)	Mean change	17.235	3.985 – 30.486	0.011*
Sedentary behavior (h/d)	5.8 (2.5)	5.4 (2.6)	Mean change	-0.358	-0.641 - -0.076	0.013*
Substance use						
Current smoking	29 (11.4%)	25 (9.8%)	OR	0.20	0.02 – 1.71	0.219†
Alcohol, 4 times/wk or more	30 (11.8%)	25 (9.8%)	OR	0.62	0.26 – 1.48	0.383†
Stress management						
Perceived stress (PSS-10; range 0-40)$	16.2 (5.8)	14.9 (5.4)	Mean change	-1.318	-1.920 - - 0.715	**<0.001***
Sleep						
Sleep problems (range 1-10)$	4.6 (2.6)	4.1 (2.6)	Mean change	-0.482	-0.803 - -0.162	0.003*
Fatigue (range 1-10)$	4.6 (2.3)	4.5 (2.4)	Mean change	-0.071	-0.371 – 0.230	0.644*
Relationships						
Social support (OSSS-3; range 3-14)§	9.5 (2.2)	9.7 (2.2)	Mean change	0.282	0.048 – 0.516	0.018*
**Medication adherence**						
SMAQ (no adherence)	137 (60.9%)	127 (53.8%)	OR	0.57	0.33 – 0.99	0.059†
**Depression**						
PHQ-8 (range 0-24)$	5.6 (4.0)	4.9 (3.8)	Mean change	-0.698	-1.104 - -0.293	**<0.001***
**HR-QoL**						
PCS (SF-12; range 0-100)§	45.6 (8.8)	46.4 (9.0)	Mean change	0.878	-0.043 – 1.798	0.062*
MCS (SF-12; range 0-100)§	45.8 (9.6)	46.7 (9.1)	Mean change	0.870	-0.249 – 1.989	0.127*

EQ-5D-5L utility values (range <0-1)§	0.80 (0.15)	0.83 (0.16)	Mean change	0.025	0.009 – 0.040	**0.002***
EQ-5D-5L overall health (range 0-100)§	71.8 (16.6)	75.3 (16.8)	Mean change	3.549	1.564 – 5.534	**<0.001***

Data shown are the available data of the subgroup 'at risk of CVD' (n=255), of the 324 participants who completed the baseline and follow-up questionnaires and attended ≥4 of 7 SEFAC sessions.
Data are mean (SD) or number of participants (%).
The effect variable shows 'mean change' for continuous variables or 'odds ratio' for dichotomous variables.
Abbreviations: SEFAC, Social Engagement Framework for Addressing the Chronic-disease-challenge; CVD, cardiovascular disease; SEMCD, Self-Efficacy for Managing Chronic Disease scale; GSES, General Self-Efficacy Scale; PESES, Physical Exercise Self-Efficacy Scale; NSES, Nutrition; OR, odds ratio; Self-Efficacy Scale; PSS-10, Perceived Stress Scale; OSSS-3, Oslo Social Support Scale; SMAQ, Short Medication Adherence Questionnaire; PHQ-8, Patient Health Questionnaire; HR-QoL, Health-related quality of life; PCS, Physical Component Summary of the SF-12; MCS, Mental Component Summary of the SF-12; SF-12, Short Form health survey; EQ-5D-5L, EuroQol-5 Dimensions-5 level
* P-value based on paired t-test; significant P-values in bold
† P-value based on McNemar test; significant P-values in bold
$ A lower score is better
§ A higher score is better
γ Odds ratio cannot be calculated due to empty cells.
Significant P-values in bold after Bonferroni correction for multiple testing was applied (P = 0.05/20 = 0.0025)
