# Supplementary material for: Evaluation of an Intervention to Promote Self-Management Regarding Cardiovascular Disease: The Social Engagement Framework for Addressing the Chronic-Disease-Challenge (SEFAC)
Source: Int J Environ Res Public Health. 2022 Oct 12;19(20):13145. doi: 10.3390/ijerph192013145 (PMC9603702; doi:10.3390/ijerph192013145)
Supplement: Supplementary file 1 [file ijerph-19-13145-s001.zip › Supplementary Table S4.pdf]

Supplementary Table S4. Satisfaction with the SEFAC intervention at follow-up (n=324)

		Total
1.	The SEFAC program was of benefit to me (% agree/strongly agree)	299 (92.3%)
2.	The SEFAC program was worth my investment in time and effort (% agree/strongly agree)	300 (92.9%)
3.	The mindfulness training stimulated me to work on a healthy lifestyle (% agree/strongly agree)	276 (85.4%)
4.	The support of volunteers stimulated me to work on a healthy lifestyle (% agree/strongly agree)	233 (74.9%)
5.	The SEFAC app stimulated me to work on a healthy lifestyle (% agree/strongly agree)	192 (75.0%)
6.	I have become more aware of my moment-to-moment physical sensations, thoughts, and emotions and I can better accept them without getting lost in them (% agree/strongly agree)	273 (84.8%)
7.	I expect that the benefits of the SEFAC program will last (% agree/strongly agree)	279 (86.9%)
8.	How satisfied are you with the SEFAC program as a whole (scale 1-10)?	8.2 (1.6)

Data shown are the available data of the 324 participants who completed the baseline and follow-up questionnaires and attended ≥4 of 7 SEFAC sessions.
Data are mean (SD) or number of participants (%)
Abbreviations: SEFAC, Social Engagement Framework for the Chronic disease challenge; SD, standard deviation
