# Supplementary material for: Evaluation of an Intervention to Promote Self-Management Regarding Cardiovascular Disease: The Social Engagement Framework for Addressing the Chronic-Disease-Challenge (SEFAC)
Source: Int J Environ Res Public Health. 2022 Oct 12;19(20):13145. doi: 10.3390/ijerph192013145 (PMC9603702; doi:10.3390/ijerph192013145)
Supplement: Supplementary file 1 [file ijerph-19-13145-s001.zip › Supplementary Table S1.pdf]

Supplementary Table S1. Overview SEFAC intervention

**Session**	**Content**
Session 1.<br>Training mind and body for well-being	• Program overview<br>• Group presentation<br>• The automatic pilot of the mind<br>• Primary and secondary suffering<br>• Mindfulness: intention, attention and attitude<br>• Recognizing the unsettled mind<br>• Settling the mind<br>• MINDFUL ATTITUDE: non judgment<br>• Positive Health model
Session 2.<br>Healthy habits	• Reviewing home assignments<br>• Habit loop<br>• Risk and protective factors<br>• Three-minute breathing space<br>• Habit change worksheet<br>• Body scan<br>• MINDFUL ATTITUDE: Beginner's mind<br>• Breathing meditation<br>• Home assignments<br>• Gratitude practice<br>• Pleasant and unpleasant experiences
Session 3.<br>Healthy mindset	• Reviewing home assignments<br>• Beliefs and mindset; fixed vs growth mindset<br>• Stages of change<br>• Readiness to change<br>• Self-compassion break<br>• Self-compassion test<br>• Sound (meditation)<br>• MINDFUL ATTITUDE: compassion and trust<br>• Home assignments
Session 4.<br>Healthy eating	• Reviewing home assignments<br>• Mindful eating<br>• Emotional eating awareness<br>• Emotional and craving eating<br>• Stress definition<br>• Perceived stress scale<br>• Self-efficacy awareness<br>• MINDFUL ATTITUDE: patience<br>• Home assignments
Session 5.<br>Healthy physical activity	• Reviewing home assignments<br>• Mindful movements<br>• Why and how stay active<br>• Physical activity awareness<br>• Setting SMART goals<br>• GROW Model<br>• Mindful walking<br>• MINDFUL ATTITUDE: non striving<br>• Home assignments
Session 6.<br>Healthy relationships	• Reviewing home assignments<br>• Why relationships are important for well-being

	• Emotional intelligence<br>• Emotions list<br>• Mindful communication<br>• STOP practice<br>• Loving kindness practice<br>• MINDFUL ATTITUDE: acceptance<br>• Mindful based living
Session 7.<br>Healthy life with chronic conditions	• Reviewing home assignments<br>• What are chronic conditions<br>• What is self-management<br>• My self-management skills<br>• Embracing vulnerability<br>• My level of resilience<br>• MINDFUL ATTITUDE: letting go and letting be<br>• Mindfulness based living
