# Supplementary material for: Evaluation of an Intervention to Promote Self-Management Regarding Cardiovascular Disease: The Social Engagement Framework for Addressing the Chronic-Disease-Challenge (SEFAC)
Source: Int J Environ Res Public Health. 2022 Oct 12;19(20):13145. doi: 10.3390/ijerph192013145 (PMC9603702; doi:10.3390/ijerph192013145)
Supplement: Supplementary file 1 [file ijerph-19-13145-s001.zip › Supplementary Table S2.pdf]

Supplementary Table S2. Baseline characteristics of the SEFAC study sample, stratified by subgroups 'History of CVD' and 'At risk of CVD' (n=352).

**Subgroup**	History of CVD (n=77)	At risk of CVD (n=275)	P-value
Age, y*	69.8 (7.9)	65.9 (7.7)	**<0.001†**
Female sex	45 (58.4%)	235 (85.5%)	**<0.001***
Study site			**<0.001***
Croatia	21 (27.3%)	128 (46.5%)	
Italy	18 (23.4%)	79 (28.7%)	
the Netherlands	38 (49.4%)	68 (24.7%)	
Other chronic conditions			
Cancer	9 (11.7%)	47 (17.1%)	0.252*
(Osteo)arthritis	31 (40.3%)	109 (39.6%)	0.921*
Pulmonary condition(s)	11 (14.3%)	37 (13.5%)	0.851*
Gastrointestinal condition(s)*	7 (9.1%)	17 (6.2%)	0.375*
Current smoking	4 (5.2%)	30 (10.9%)	0.134*
Alcohol use ≥4 times/wk	10 (13.0%)	33 (12.0%)	0.815*
Aerobic physical activity <150 min/wk	24 (31.2%)	121 (44.0%)	**0.043***
Fruit <3 servings/d*	64 (83.1%)	248 (90.5%)	0.068*
Vegetables <3 servings/d†	69 (89.5%)	257 (94.1%)	0.155*
Overweight (BMI ≥25 kg/m^2)	51 (66.2%)	175 (63.6%)	0.674*
Current depression (PHQ-8 ≥10)	17 (22.1%)	47 (17.1%)	0.316*
Moderate-high perceived stress (PSS-10 ≥14)	57 (74.0%)	188 (68.4%)	0.340*
Living alone	32 (41.6%)	91 (33.1%)	0.168*
Education			0.402*
Primary or no education	11 (14.3%)	43 (15.6%)	
Secondary	41 (53.2%)	123 (44.7%)	
Tertiary or higher	25 (32.5%)	109 (39.6%)	
Low income (decile 1 and 2)‡	13 (17.1%)	31 (11.8%)	0.229*
Migration background	15 (19.5%)	37 (13.5%)	0.188*

Data are mean (SD) or number of participants (%).
Missing items: * n=1; † n=3; ‡ n=14
Abbreviations: CVD, cardiovascular disease; BMI, body mass index; PHQ-8, Patient Health Questionnaire; PSS-10, Perceived Stress Scale; SD, standard deviation
* P-value based on chi-squared test; significant P-values in bold
† P-value based on independent student's T-test; significant P-values in bold
