# Supplementary material for: Evaluation of an Intervention to Promote Self-Management Regarding Cardiovascular Disease: The Social Engagement Framework for Addressing the Chronic-Disease-Challenge (SEFAC)
Source: Int J Environ Res Public Health. 2022 Oct 12;19(20):13145. doi: 10.3390/ijerph192013145 (PMC9603702; doi:10.3390/ijerph192013145)
Supplement: Supplementary file 1 [file ijerph-19-13145-s001.zip › Supplementary Table S3a_final.pdf]

Supplementary Table S3a. Effects of the SEFAC intervention, stratified by subgroups 'History of CVD' and 'At risk of CVD', showing subgroup 'History of CVD' (n=69)

**Outcomes**	Baseline	Follow-up (6 month)	Effect variable	Estimate	Confidence interval	P-value#
**Self-efficacy**						
SEMCD (range 1-10) §	6.7 (1.7)	7.0 (1.7)	Mean change	0.372	-0.043 – 0.787	0.078*
GSES (range 10-40) §	29.9 (6.3)	31.9 (5.8)	Mean change	2.000	0.655 – 3.345	0.004*
PESES (range 5-20) §	13.2 (3.8)	13.8 (4.0)	Mean change	0.522	0.461 – 1.505	0.293*
NSES (range 5-20) §	14.0 (3.2)	15.0 (3.4)	Mean change	1.000	0.194 – 1.806	0.016*
**Health behaviors**						
Nutrition						
Fruit ≥3 portions/d	12 (17.4%)	11 (15.9%)	OR	0.83	0.25 – 2.73	1.000†
Vegetables, ≥3 portions/d	7 (10.3%)	8 (11.6%)	OR	1.20	0.37 – 3.93	1.000†
Physical activity						
Stretching/strengthening (min/wk)	44.1 (56.9)	39.5 (58.0)	Mean change	-4.545	-21.023 – 11.932	0.584*
Aerobic exercise (min/wk)	183.9 (105.7)	180.4 (106.7)	Mean change	-3.478	-25.494 – 18.537	0.754*
Sedentary behavior (h/d)	6.0 (2.6)	5.4 (2.5)	Mean change	-0.510	-1.155 – 0.136	0.120*
Substance use						
Current smoking	4 (5.8%)	4 (5.8%)	OR	-γ	-γ	1.000†
Alcohol, 4 times/wk or more	10 (14.5%)	10 (14.5%)	OR	1.00	0,14 – 7,10	1.000†
Stress management						
Perceived stress (PSS-10; range 0-40)$	16.5 (6.4)	15.6 (5.4)	Mean change	-0.841	-2.107 – 0.426	0.190*
Sleep						
Sleep problems (range 1-10)$	5.1 (2.7)	4.9 (2.6)	Mean change	-0.071	-0.767 – 0.419	0.560*
Fatigue (range 1-10)$	5.4 (2.2)	5.2 (2.3)	Mean change	-0.174	-0.737 – 0.389	0.540*
Relationships						
Social support (OSSS-3; range 3-14)§	9.2 (2.3)	9.7 (2.6)	Mean change	0.529	0.010 – 1.049	0.046*
**Medication adherence**						
SMAQ (no adherence)	42 (60.9%)	41 (59.4%)	OR	0.90	0.37 – 2.21	1.000†
**Depression**						
PHQ-8 (range 0-24)$	6.2 (4.8)	5.3 (4.5)	Mean change	-0.899	-1.622 – -0.175	0.016*
**HR-QoL**						
PCS (SF-12; range 0-100)§	40.9 (9.3)	43.6 (8.1)	Mean change	2.217	0.482 – 3.952	0.013*

MCS (SF-12; range 0-100)§	44.2 (11.5)	46.3 (9.7)	Mean change	2.209	0.080 – 4.339	0.042*
EQ-5D-5L utility values (range <0-1)§	0.77 (0.15)	0.79 (0.17)	Mean change	0.025	0.012 – 0.063	0.177*
EQ-5D-5L overall health (range 0-100)§	67.5 (16.5)	68.5 (19.0)	Mean change	1.073	-2.409 - -4.554	0.541*

Data shown are the available data of the subgroup 'History of CVD' (n=69) of the 324 participants who completed the baseline and follow-up questionnaires and attended ≥4 of 7 SEFAC sessions.
Data are mean (SD) or number of participants (%).
The effect variable shows 'mean change' for continuous variables or 'odds ratio' for dichotomous variables.
Abbreviations: SEFAC, Social Engagement Framework for Addressing the Chronic-disease-challenge; CVD, cardiovascular disease; SEMCD, Self-Efficacy for Managing Chronic Disease scale; GSES, General Self-Efficacy Scale; PESES, Physical Exercise Self-Efficacy Scale; NSES, Nutrition; OR, odds ratio; Self-Efficacy Scale; PSS-10, Perceived Stress Scale; OSSS-3, Oslo Social Support Scale; SMAQ, Short Medication Adherence Questionnaire; PHQ-8, Patient Health Questionnaire; HR-QoL, Health-related quality of life; PCS, Physical Component Summary of the SF-12; MCS, Mental Component Summary of the SF-12; SF-12, Short Form health survey; EQ-5D-5L, EuroQol-5 Dimensions-5 level
* P-value based on paired t-test; significant P-values in bold
† P-value based on McNemar test; significant P-values in bold
$ A lower score is better
§ A higher score is better
γ Odds ratio cannot be calculated due to empty cells.
Significant P-values in bold after Bonferroni correction for multiple testing was applied (P = 0.05/20 = 0.0025)
