# Supplementary material for: Evaluation of an Intervention to Promote Self-Management Regarding Cardiovascular Disease: The Social Engagement Framework for Addressing the Chronic-Disease-Challenge (SEFAC)
Source: Int J Environ Res Public Health. 2022 Oct 12;19(20):13145. doi: 10.3390/ijerph192013145 (PMC9603702; doi:10.3390/ijerph192013145)
Supplement: Supplementary file 1 [file ijerph-19-13145-s001.zip › Supplementary Table S3c.pdf]

Supplementary Table S3c. Comparing mean change in outcome measures of the SEFAC intervention in subgroup ‘History of CVD (n=69)’ and ‘At risk of CVD (n=255)’

	History of CVD (n=69)	At risk of CVD (n=255)	
**Outcomes**	Estimate		P-value[#]
**Self-efficacy**			
SEMCD (range 1-10) [§]	0.372	0.309	0.143[α]
GSES (range 10-40) [§]	2.000	1.262	0.604[α]
PESES (range 5-20) [§]	0.522	0.537	0.737[α]
NSES (range 5-20) [§]	1.000	0.710	0.939[α]
**Health behaviors**			
Nutrition			
Fruit ≥3 portions/d	0.83	1.29	0.537[β]
Vegetables, ≥3 portions/d	1.20	1.30	0.914[β]
Physical activity			
Stretching/strengthening (min/wk)	-4.545	-5.374	0.631[α]
Aerobic exercise (min/wk)	-3.478	17.235	0.048[α]
Sedentary behavior (h/d)	-0.510	-0.358	0.224[α]
Substance use			
Current smoking	-[γ]	0.20	-[γ]
Alcohol, 4 times/wk or more	1.00	0.62	0.658[β]
Stress management			
Perceived stress (PSS-10; range 0-40)[$]	-0.841	-1.318	0.725[α]
Sleep			
Sleep problems (range 1-10)[$]	-0.071	-0.482	0.555[α]
Fatigue (range 1-10)[$]	-0.174	-0.071	0.928[α]
Relationships			
Social support (OSSS-3; range 3-14)[§]	0.529	0.282	0.202[α]
**Medication adherence**			
SMAQ (no adherence)	0.90	0.57	0.399[β]
**Depression**			
PHQ-8 (range 0-24)[$]	-0.899	-0.698	0.258[α]
**HR-QoL**			
PCS (SF-12; range 0-100)[§]	2.217	0.878	0.999[α]
MCS (SF-12; range 0-100)[§]	2.209	0.870	0.903[α]
EQ-5D-5L utility values (range <0-1)[§]	0.025	0.025	0.019[α]
EQ-5D-5L overall health (range 0-100)[§]	1.073	3.549	0.546[α]

Data shown are the available data of the 324 participants who completed the baseline and follow-up questionnaires and attended ≥4 of 7 SEFAC sessions, divided in subgroups ‘History of CVD’ and ‘At risk of CVD’. The estimate shows ‘mean change’ for continuous variables or ‘odds ratio’ for dichotomous variables. Abbreviations: SEFAC, Social Engagement Framework for Addressing the Chronic-disease-challenge; CVD, cardiovascular disease; SEMCD, Self-Efficacy for Managing Chronic Disease scale; GSES, General Self-Efficacy

Scale; PESES, Physical Exercise Self-Efficacy Scale; NSES, Nutrition Self-Efficacy Scale; PSS-10, Perceived Stress Scale; OSSS-3, Oslo Social Support Scale; SMAQ, Short Medication Adherence Questionnaire; PHQ-8, Patient Health Questionnaire; HR-QoL, Health-related quality of life; PCS, Physical Component Summary of the SF-12; MCS, Mental Component Summary of the SF-12; SF-12, Short Form health survey; EQ-5D-5L, EuroQol-5 Dimensions-5 level

[α] P-values based on independent t-tests

[β] P-values based on z-tests

[$] A lower score is better

[§] A higher score is better

[γ] Odds ratio cannot be calculated due to empty cells.

[#] Significant P-values in bold after Bonferroni correction for multiple testing was applied (P = 0.05/20 = 0.0025)
